# Mechanisms of the action of adenine on anti‐allergic effects in mast cells

**DOI:** 10.1002/iid3.200

**Published:** 2017-11-01

**Authors:** Toru Hosoi, Shinsuke Ino, Fumie Ohnishi, Kenichi Todoroki, Michiko Yoshii, Mai Kakimoto, Christa E. Müller, Koichiro Ozawa

**Affiliations:** ^1^ Department of Pharmacotherapy Graduate School of Biomedical and Health Sciences Hiroshima University 1‐2‐3 Kasumi, Minami‐ku Hiroshima 734‐8551 Japan; ^2^ PharmaCenter Bonn Pharmaceutical Institute, Pharmaceutical Chemistry I, University of Bonn An der Immenburg 4 D‐53121 Bonn Germany

**Keywords:** adenine, mast cells, allergy

## Abstract

**Introduction:**

Mast cells play an important role in allergic responses.

**Methods:**

We herein demonstrated the mechanisms of inhibitory effect of adenine on IgE/antigen‐induced degranulation and TNF‐α release in mast cells.

**Results:**

We found that these effects were dependent on the amino group of adenine because purine only weakly inhibited degranulation. Adenine also inhibited Ca^2+^ ionophore‐ and thapsigargin‐induced degranulation, however, this inhibitory effect was weaker than that of the antigen. Therefore, the inhibitory effects of adenine on degranulation may be mediated before as well as after the Ca^2+^ raise under the antigen stimulus. Adenine inhibited antigen‐induced Syk and the subsequent induction of AKT and ERK activation under FcϵRI‐mediated signal. Adenine also attenuated antigen‐induced increase in Ca^2+^. Furthermore, adenine inhibited IgE/antigen‐induced IKKα/β activation, which is involved in degranulation. Finally, adenine protected mice against anaphylactic allergic responses in vivo.

**Conclusions:**

The present study revealed a key role of adenine in the attenuation of allergic responses through the inhibition of Syk‐mediated signal transduction and IKK‐mediated degranulation.

## Introduction

Mast cells play an important role in allergic responses. They store histamine in cytoplasmic secretory granules and are activated by antigen/IgE‐mediated cross linking of the FcϵRI receptor. FcϵRI is a heterotetrameric receptor that is composed of an IgE‐binding α subunit, four‐transmembrane β subunits, and two disulfide‐bonded γ subunits 1. The common motif of the immunoreceptor tyrosine‐based activation motif (ITAM), which exists in the β and γ subunits, is involved in the cell signaling. The crosslinking of FcϵRI‐bound IgE with an antigen activates Lyn, which, in turn, phosphorylates ITAM and activates Syk. The activation of Syk induces the release of histamine by phosphorylating phospholipase C (PLC)‐γ, which hydrolyze phosphatidylinositol 4,5‐bisphosphate (PIP_2_) into diacylglycerol (DAG) and inositol 1,4,5‐triphosphate (IP_3_). IP_3_ acts on the endoplasmic reticulum and increases intracellular Ca^2+^ levels, while DAG activates protein kinase C (PKC). Synergistic degranulation is induced by the generation of these two second messengers. Soluble N‐ethylmaleimide‐sensitive fusion factor attachment protein receptor (SNARE) proteins play a role in exocytosis by promoting vesicle fusion with the plasma membrane 2. A previous study reported that activated mast cells induced the phosphorylation of IκB kinase (IKK) β, which promoted degranulation through the phosphorylation of synaptosomal‐associated protein 23 (SNAP‐23) 3. Upon its activation, Syk also phosphorylates various downstream signaling molecules and propagates signaling pathways through the activation of phosphatidylinositol‐3‐OH kinase (PI3K) and mitogen‐activated protein kinase (MAPK) 4, 5. Many of these kinases are known to be involved in the production of histamine as well as cytokines, such as TNFα.

Adenosine is a well‐known signaling molecule that exerts biological functions through A_1_, A_2A_, A_2B_, and A_3_ receptors in various cells and organs, which are considered to be potential therapeutic targets 6. Adenosine is released by various cells during ischemia and inflammation and activates mast cells. The important role of adenosine in histamine release in mast cells has already been reported 7, 8. Previous studies demonstrated that adenosine amplified the release of histamine from mast cells 7, 8. In contrast to adenosine, adenine was recently found to be a signaling molecule. The adenine receptor was identified from the orphan rat G protein‐coupled receptor (GPCR) and shown to be a member of the MAS‐value gene (Mrg) receptor family, which is expressed on various tissues such as dorsal root ganglia, lung, hypothalamus, peripheral blood leukocytes, and ovaries 9. Adenine was previously reported to promote purkinje cell survival 10, and was shown to increase rat hepatic stellate cell differentiation 11. Therefore, adenine may act physiologically as a signaling molecule in mammals. We herein revealed a unique function of adenine, which exhibited anti‐allergic activity by inhibiting several protein kinases involved in the release of histamine.

## Materials and Methods

### Materials and reagents

Monoclonal anti‐dinitrophenyl (DNP) antibody, DNP‐BSA, and A23187 were obtained from Sigma (MO). Thapsigargin was obtained from Wako Pure Chemical Industries, Ltd. (Japan).

### Cell culture

The rat basophilic leukemia mast cell line, RBL‐2H3, was cultured in RPMI medium containing 15 % fetal calf serum, 0.29 mg/mL l‐glutamine, 100 U/mL of penicillin G, and 100 μg/mL of streptomycin (Nacalai Tesque, Japan). These cells were kept at 37 °C in 5 % CO_2_/95 % air.

### Measurement of degranulation

We measured β hexosaminidase activity as an index of degranulation as described previously 12. RBL‐2H3 cells were plated on a 96‐well plate and sensitized with DNP‐IgE (0.5 µg/mL). After an overnight incubation, the medium was replaced with Siraganian Buffer (119 mM NaCl, 5 mM KCl, 0.4 mM MgCl_2_, 5.6 mM glucose, 25 mM PIPES, 1 mM CaCl_2_, and 0.1 % BSA, pH 7.2) and incubated with adenine or adenosine for 30 min. The cells were then stimulated with DNP‐BSA (20 ng/mL), A23187, or thapsigargin for 15 min at 37 °C. The reaction was stopped by placing the plate on ice, followed by centrifuging at 4000 rpm for 1 min at 4 °C, and supernatants were then collected. The supernatants were reacted with 10 µL of p‐NAG (1 mM p‐nitrophenyl‐N‐acetyl‐β‐d‐glucosaminide) for 1 h at 37 °C and the reaction was stopped by adding 0.1 M Na_2_CO_3_/NaHCO_3_ (pH 10). Absorbance at 415 nm was measured using a 96‐well microplate reader (VERSA max tunable microplate reader, Molecular Devices, CA).

### Measurement of histamine

The histamine measurement released form RBL‐2H3 mast cells are conducted using EIA method using histamine enzyme immunoassay kit (A05890. 96 wells, Bertin Pharma, France).

### Western blot analysis

Western blotting was performed as described previously 13. Briefly, cells were washed with ice‐cold PBS and lysed in buffer containing 10 mM HEPES‐NaOH (pH 7.5), 150 mM NaCl, 1 mM EGTA, 1 mM Na_3_VO_4_, 10 mM NaF, 10 μg/mL aprotinin, 10 μg/mL leupeptin, 1 mM phenylmethylsulfonyl fluoride (PMSF), and 1% NP‐40 for 20 min. The lysates were centrifuged at 15,000 rpm for 20 min at 4 °C, and supernatants were collected. Samples were boiled in Laemmli buffer for 3 min, fractionated by sodium dodecylsulfate‐polyacrylamide gel electrophoresis (SDS‐PAGE), and transferred at 4°C to nitrocellulose membranes. These membranes were then incubated with anti‐p‐ERK (Cell signaling 9101 [0.056 mg/mL]; 1:1000), anti‐p‐Akt (Thr 308, Cell signaling 9275 [0.2 mg/mL]; 1:1000), anti‐Akt (Cell signaling 9272 [0.16 mg/mL]; 1:1000), anti‐phospho Tyrosine (Upstate 05‐321 [1 mg/mL]; 1:1000), anti‐Syk (Santa Cruz sc‐1077 [0.2 mg/mL]; 1:1000), and anti‐GAPDH (Chemicon MAB374 [1 mg/mL]; 1:1000) antibodies, followed by an anti‐horseradish peroxidase‐linked antibody. Peroxidase binding was detected by chemiluminescence using an enhanced chemiluminescence system (GE Healthcare).

### Immunoprecipitation

Cells were lysed in lysis buffer (10 mM HEPES‐NaOH [pH 7.5], 150 mM NaCl, 1 mM EGTA, 1 mM Na_3_VO_4_, 10 mM NaF, 10 µg/mL aprotinin, 10 µg/mL leupeptin, 1 mM PMSF, and 0.1% NP‐40) and samples were homogenized using a 21G needle. The lysates were centrifuged at 15,000 rpm for 15 min at 4°C, and the supernatants were collected. The supernatants were pre‐incubated with Protein G‐sepharose and normal IgG for 1 h at 4 °C. An antibody was incubated with Protein G‐sepharose and rotated at 4 °C for 1 h. After washing with BSA‐lysis buffer, Protein G‐sepharose was added to the cell lysates and rotated at 4 °C for 4 h. Immunoprecipitates were washed three times with lysis buffer and boiled for 2 min in Laemmli buffer. The samples were centrifuged at 10,000 rpm for 30 sec at 4°C and the supernatants were used for SDS–PAGE.

### ELISA analysis for TNF‐α measurements

RBL‐2H3 cells were sensitized with DNP‐IgE (0.5 µg/mL). After an overnight incubation, the cells were treated with the each of the drugs for 30 min, and then stimulated with DNP‐BSA (20 ng/mL) for 12 h at 37 °C. The medium was centrifuged at 800×*g* for 10 min at 4 °C and the supernatant was used for the ELISA assay. We used the DuoSet^®^ ELISA Development kit (R&D Systems, MN) for measurements.

### Ca^2+^ measurements

RBL‐2H3 cells were grown on cover glass and sensitized with DNP‐IgE (0.5 µg/mL) overnight. The cells were then washed with Siraganian Buffer (119 mM NaCl, 5 mM KCl, 0.4 mM MgCl_2_, 5.6 mM glucose, 25 mM PIPES, 1 mM CaCl_2_, and 0.1 % BSA, pH 7.2) and incubated with adenine + fura‐2/AM solution for 30 min at 37 °C. The cells were set on the cell chamber and stimulated with DNP‐BSA. Ca^2+^ measurements were conducted using ARGUS‐HISCA150 and the ratio of the excitation wavelength at 340/380 was analyzed.

### Passive cutaneous anaphylaxis (PCA) experiment

C57BL/6 mice were used in the PCA experiment, which was performed as described previously 14, 15 with slight modifications. Briefly, an anti‐DNP‐IgE antibody (20 µg/mL, 10 µL) was administered subcutaneously into the ear for sensitization. In the control experiment, PBS was injected instead of the anti‐DNP‐IgE antibody. Forty‐eight hours after the sensitization, adenine (50 mg/kg) was injected intraperitoneally. Thirty minutes after this administration, DNP‐BSA (1 mg/mL) in Evans blue (0.5%) were injected intravenously (10 mL/kg). Mice were killed 30 min after the injection and both of the ears were dissolved in KOH (1 M, 1 mL) and incubated at 37 °C overnight with shaking. In the preliminary experiments, we measured weight of mice ears and the difference of individual mice ears were 0.31 g ± 0.0081 (*n* = 7). The samples were then dissolved in 9 mL of acetone and 0.2 M phosphate (13:5) buffer with shaking. The samples were left to stand for 2 min, the supernatant was then centrifuged at 4000 rpm for 10 min, and its optical density was measured at 620 nm. All animals experimental methods were carried out in accordance with the NIH Guide for Care and Use of Laboratory Animals and the ethical approval had been acquired by the animal care and use committee at Hiroshima University.

### Statistics

Results are expressed as the means ± S.E. Statistical analyses were performed using a paired *t*‐test, Dunnett's test, or Bonferroni.

## Results

### The effect of adenine on allergic responses

**Figure 1:**
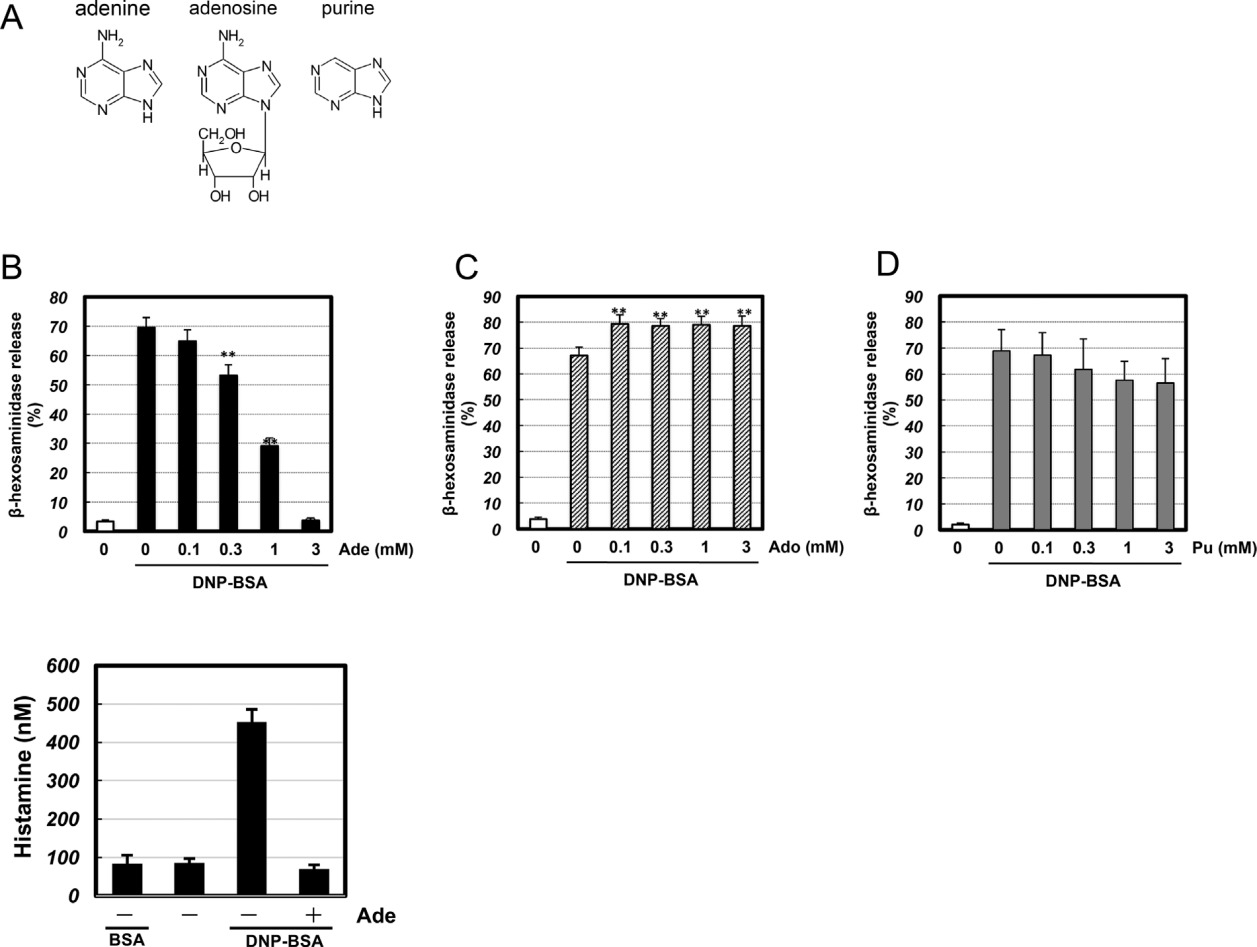
Adenine but not adenosine or purine has anti‐allergic property. (A) The chemical structural formulae of adenine, adenosine, and purine. RBL‐2H3 cells were pre‐treated with (B) adenine (Ade; 0.1–3 mM) *n* = 11, (C) adenosine (Ado; 0.1–3 mM) *n* = 3, or (D) purine (Pu; 0.1–3 mM) *n* = 3, for 30 min, and then stimulated with antigen (20 ng/mL DNP‐BSA) for 15 min. Absorbance was measured with a microplate reader at 415 nm. **p < 0.01 versus the DNP‐BSA stimulus. (E) Histamine measurement by EIA. RBL‐2H3 cells were pre‐treated with adenine (Ade; 3 mM) for 30 min, and then stimulated with antigen (20 ng/mL DNP‐BSA) for 15 min. ***p < 0.001 versus the DNP‐BSA stimulus.

**Figure 2:**
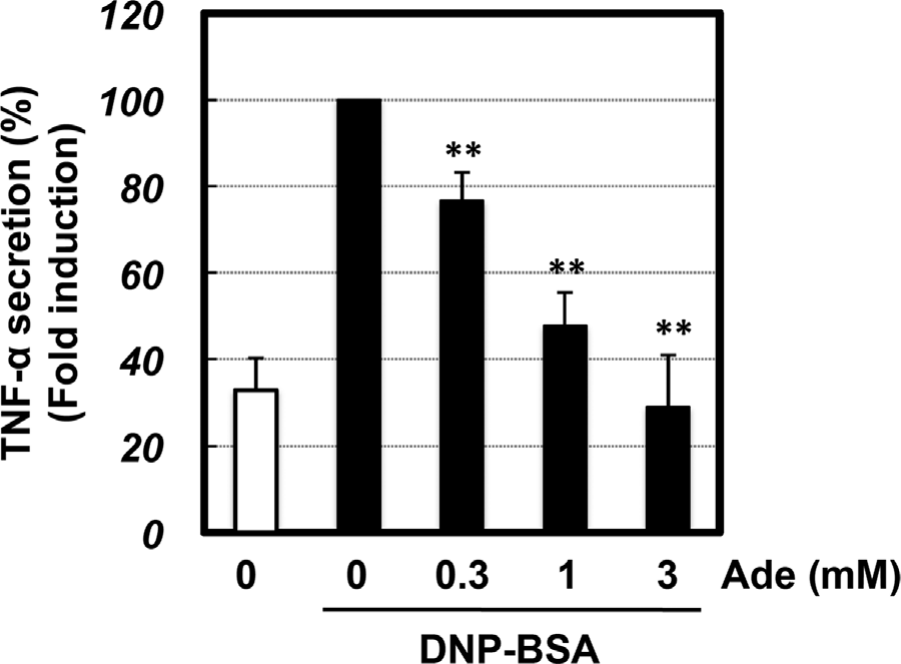
Adenine inhibited antigen‐induced TNFα release. RBL‐2H3 cells were pre‐treated with adenine (Ade; 0.3–3 mM) for 30 min and then stimulated with an antigen (20 ng/mL DNP‐BSA) for 12 h. The ELISA assay for TNFα measurement was then performed. **p < 0.01 versus the DNP‐BSA stimulus. *n* = 5.

**Figure 3:**
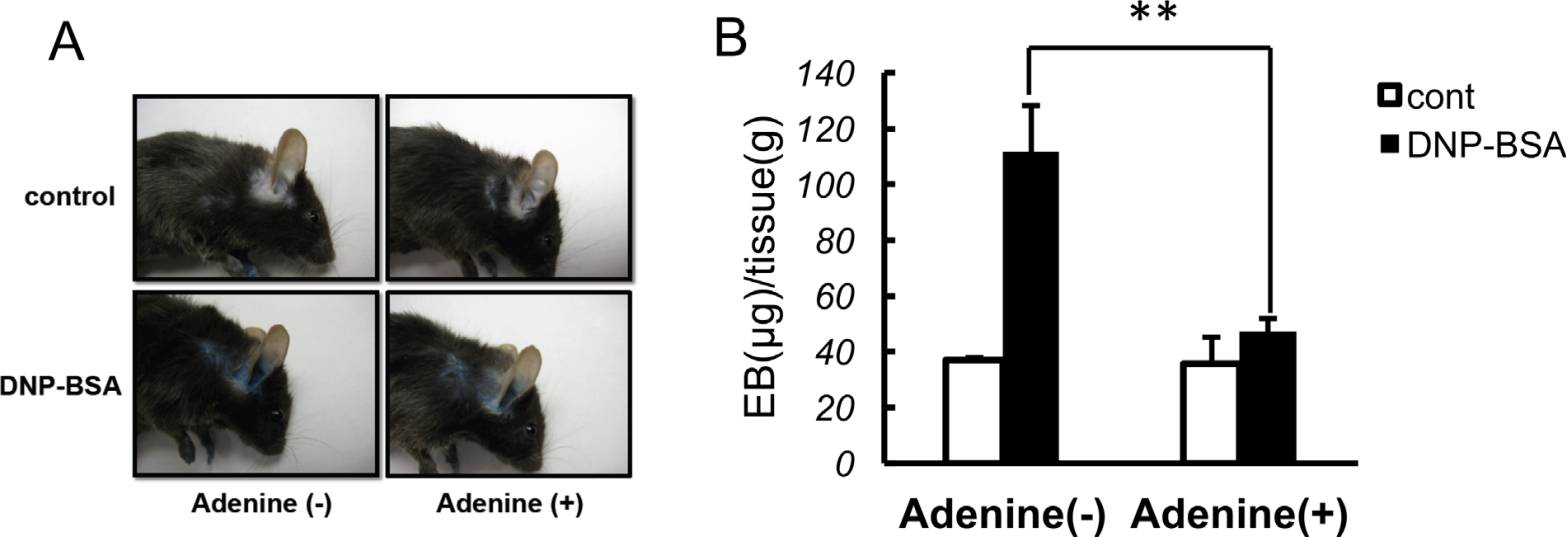
Adenine attenuated allergic response in the in vivo mice model. (A) Inhibitory effects of adenine on passive cutaneous anaphylaxis (PCA) reactions in mice. C57BL/6J mice were sensitized with anti DNP‐IgE intradermally in the ear 48 h before the DNP‐BSA treatment. Adenine (50 mg/kg, i.p.) was injected 30 min before the DNP‐BSA stimulus. Mice were stimulated with DNP‐BSA mixed with Evans blue dye for 30 min and sacrificed. Dye leakage into ear tissues was quantified. (B) Absorbance of PCA analysis was measured with a microplate reader at 620 nm. The mass of Evans blue dye (µg) per g of ear tissue were calculated. *p < 0.05 versus Adenine (+) + DNP‐BSA. *n* = 4–10.

We treated the RBL‐2H3 mast cell line with adenine and then measured IgE/antigen‐induced degranulation to determine whether adenine inhibited mast cell degranulation. Mast cell degranulation was assessed by measuring the activity of β‐hexosaminidase, a marker of histamine‐containing granules. As shown in Figure 1B, adenine markedly inhibited of IgE/antigen‐induced degranulation in a dose‐dependent manner. We also observed inhibition of histamine release by adenine in RBL‐2H3 mast cells (Fig. 1E). Furthermore, the release was not observed when the cells were treated with BSA instead of DNP‐BSA, confirming its specificity (Fig. 1E). The effects of adenine were not due to cytotoxic effects because an increase in LDH leakage was not detected (Fig. S1). On the other hand, purine only weakly inhibited IgE/antigen‐induced degranulation (Fig. 1D). These results suggest that the amino group of adenine play an important role in inhibiting degranulation. In contrast, adenosine has been shown to activate mast cells and enhance IgE/antigen‐induced degranulation 16. A3 adenosine receptor was reported to express in the RBL‐2H3 mast cells 17. We herein confirmed that adenosine enhanced IgE/antigen‐induced degranulation in the RBL‐2H3 cell line (Fig. 1C). Therefore, in contrast to adenosine, adenine inhibited IgE/antigen‐induced degranulation. We further investigated whether adenine inhibited IgE/antigen‐induced TNFα production. We sensitized mast cells with DNP‐specific IgE, then stimulated DNP‐BSA for 12 h, and measured TNFα release by ELISA. We observed a threefold increase in TNFα production by IgE/antigen, which was completely inhibited by adenine (Fig. 2). These results indicated that adenine had inhibitory effects on IgE/antigen‐induced degranulation and TNFα release. To elucidate in vivo significance of these results, we performed a passive cutaneous anaphylaxis (PCA) analysis to investigate whether adenine protects mice against anaphylactic allergic responses. We investigated the effects of adenine on the PCA reaction evoked by the antigen‐antibody reaction. Mice were sensitized by injecting anti‐DNP‐IgE into the ear through intradermal injection. We then injected Evans blue dye and analyzed leakage of the dye in the ear. We confirmed that BSA plus Evans blue dye injection did not cause the leakage of the dye in the preliminary experiments (Fig. S2). On the other hand, the increase in leakage of the Evans blue dye was detected after the DNP‐BSA plus Evans blue dye injection (Fig. 3). The leakage of the Evans blue dye was significantly attenuated when adenine was injected prior to the antigen‐antibody reaction (Fig. 3). These results suggested that adenine inhibited the PCA reaction in vivo.

### Adenine inhibited antigen‐induced Syk and the subsequent induction of AKT and ERK activation under the IgE/antigen stimulus

**Figure 4:**
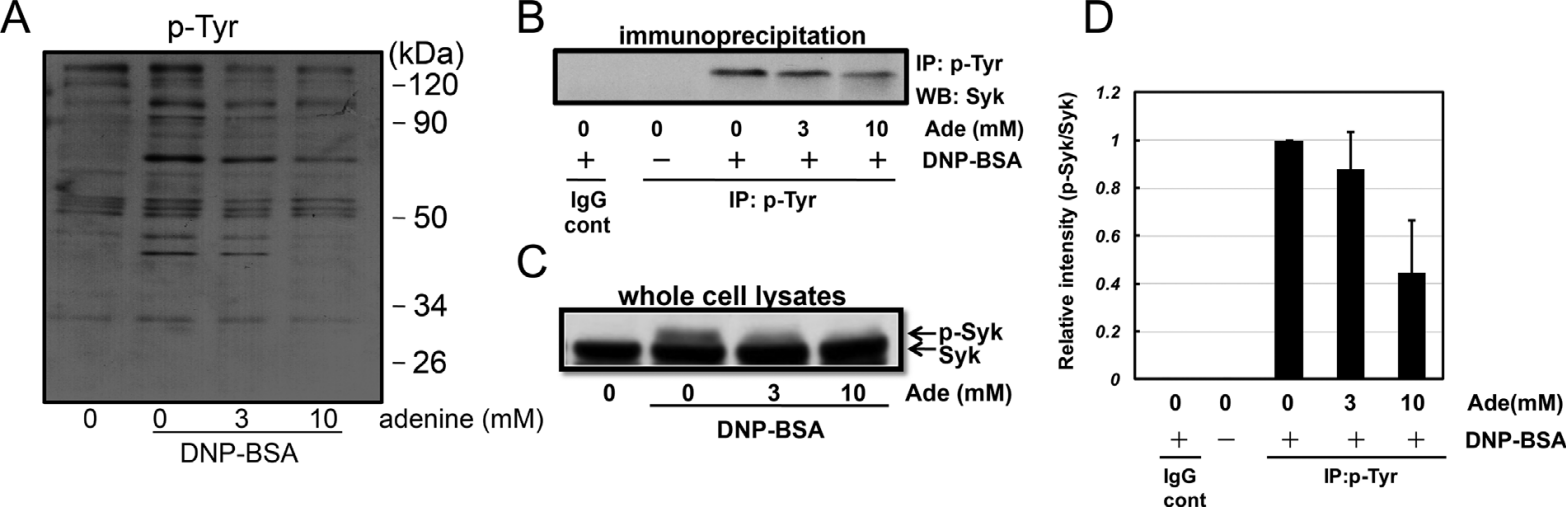
Adenine inhibited antigen‐induced Syk phosphorylation. RBL‐2H3 cells were pre‐treated with adenine (3 and 10 mM) for 30 min, and then stimulated with DNP‐BSA (20 ng/mL) for 5 min. (A) A Western blotting analysis was performed using a specific antibody for phospho‐tyrosine. Adenine inhibited several phospho‐tyrosine proteins induced by the antigen. (B) Immunoprecipitation was performed using a specific antibody for phospho‐tyrosine. A Western blotting analysis was performed using specific antibodies for Syk. (C) Western blotting was performed using whole cell lysates with a specific antibody for Syk. (D) A densitometric analysis of Syk phosphorylation using image analyzing software. The data were expressed as relative intensity of p‐SKY/total SYK. *n* = 3.

**Figure 5:**
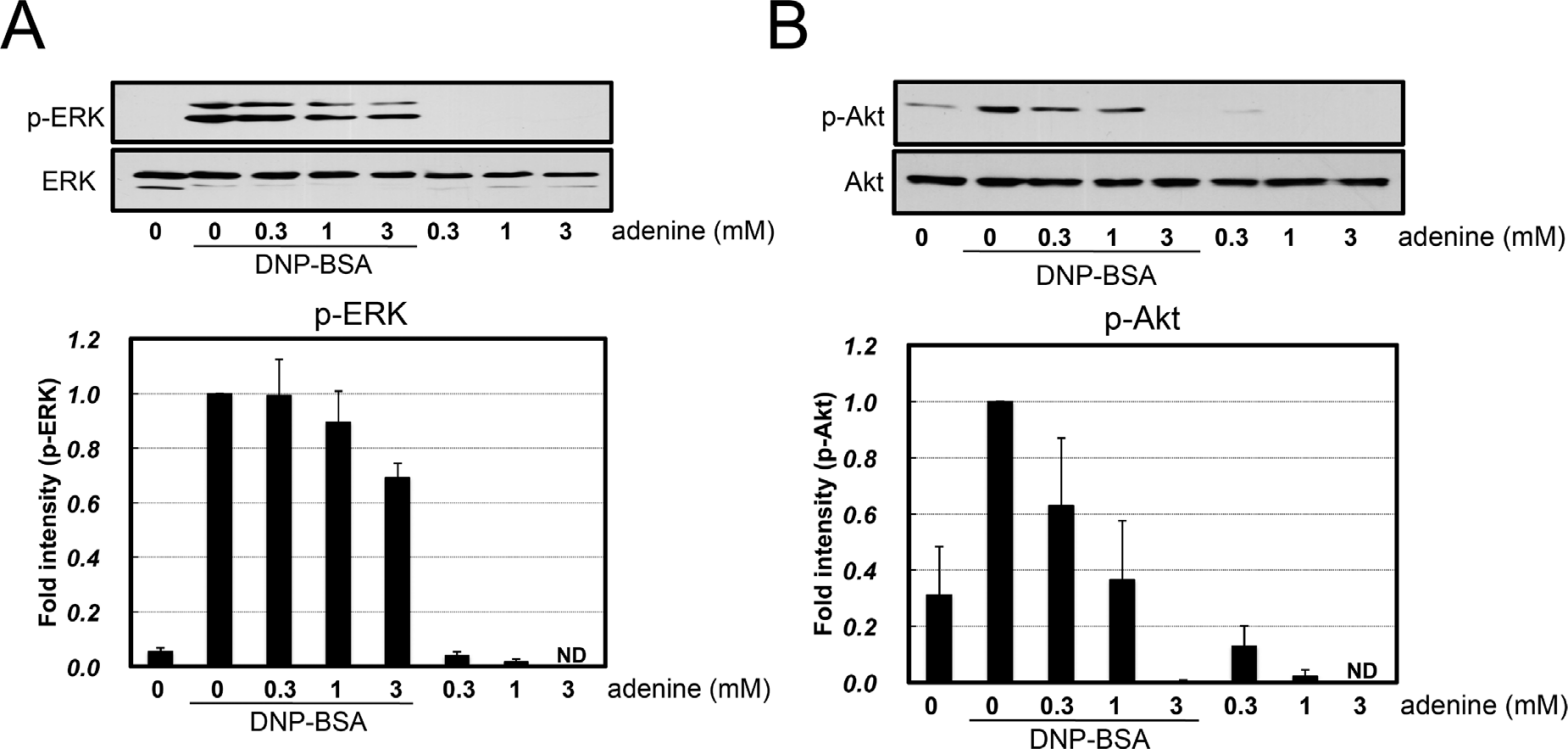
Adenine inhibited antigen‐induced ERK/Akt phosphorylation. A Western blotting analysis was performed using specific antibodies for (A) phospho‐ERK (Thr 202/Tyr 204), (B) phospho‐Akt (Thr 308), and GAPDH. A densitometric analysis of ERK and Akt phosphorylation using image analyzing software. *n* = 4.

To elucidate the mechanisms underlying the inhibitory effects of adenine on IgE/antigen‐induced degranulation, we measured total cellular tyrosine phosphorylation in adenine‐treated cells. The IgE/antigen stimulus increased the levels of several tyrosine phosphorylated proteins, which were dose‐dependently inhibited by adenine (Fig. 4A). Therefore, adenine may inhibit IgE/antigen‐induced protein phosphorylation, thereby attenuating degranulation. Among the several tyrosine‐phosphorylated proteins identified, one of the major bands at approximately 70 kDa was markedly attenuated by adenine. Syk is a 72 kDa protein, that plays a central role in IgE/antigen‐induced signaling 18, 19. Thus, we investigated whether adenine inhibited the activation of Syk. Cells were lysed and subjected to immunoprecipitation with an anti‐phospho‐tyrosine antibody and the immunoprecipitated samples were analyzed using a Syk antibody. Adenine dose‐dependently inhibited IgE/antigen‐induced Syk phosphorylation (Fig. 4B). Phosphorylated Syk was also detected as a slowly migrating band by a Western blotting analysis using whole cell lysates after the IgE/antigen stimulus, and was dose‐dependently inhibited by adenine (Fig. 4C). Therefore, adenine may inhibit IgE/antigen‐induced Syk activation. Syk is known to be activated by its phosphorylation and phosphorylates various signaling molecules downstream of Syk 18, 19. The activation of Syk has been shown to induce downstream signaling pathways such as MAP kinase and PI3K/Akt 4, 5. Thus, we examined the effects of adenine on IgE/antigen‐induced ERK and Akt phosphorylation. As shown in Figure 5, adenine dose‐dependently inhibited the phosphorylation of ERK and Akt in IgE/antigen‐sensitized mast cells. These results suggested that adenine inhibited antigen‐induced Syk and the subsequent induction of AKT and ERK activation under the IgE/antigen stimulus.

### Adenine attenuated antigen‐induced increase in Ca^2+^ levels

**Figure 6:**
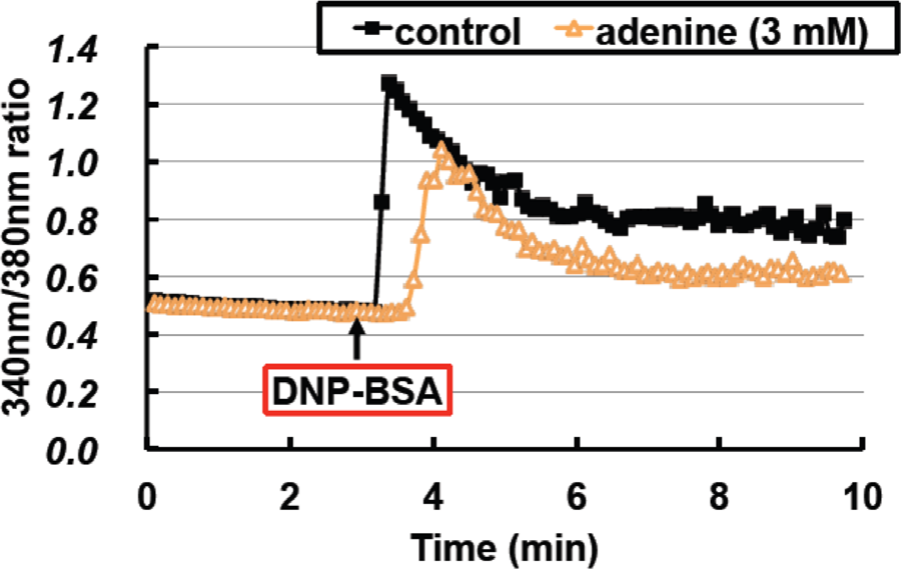
Adenine inhibited IgE‐induced Ca^2+^ mobilization. RBL‐2H3 cells were treated with fura‐2/AM in the presence or absence of adenine (3 mM) for 30 min, and then stimulated with DNP‐BSA (20 ng/mL). Intracellular levels of Ca^2+^ were determined by ARGUS‐HISCA.

The increase in Ca^2+^ levels in response to an antigen stimulus involves a biphasic process; that is, IP_3_ binds to the endoplasmic reticulum membrane IP_3_ receptor, causing the rapid transient release of Ca^2+^ from endoplasmic reticulum stores and a sustained influx of extracellular Ca^2+^ across the plasma membrane. Since adenine inhibited the activation of Syk, which increases IP_3_ levels, we determined whether adenine inhibited the increase induced in Ca^2+^ levels by the antigen stimulus. RBL‐2H3 cells were pre‐treated with adenine for 30 min and stimulated with DNP‐BSA, and Ca^2+^ levels were then measured. Transient increases in Ca^2+^ levels were observed in control cells. On the other hand, this transient increase was attenuated by adenine (Fig. 6). Therefore, adenine may inhibit the rapid transient release of Ca^2+^.

### Adenine inhibited Ca^2+^ ionophore‐ and thapsigargin‐induced degranulation

**Figure 7:**
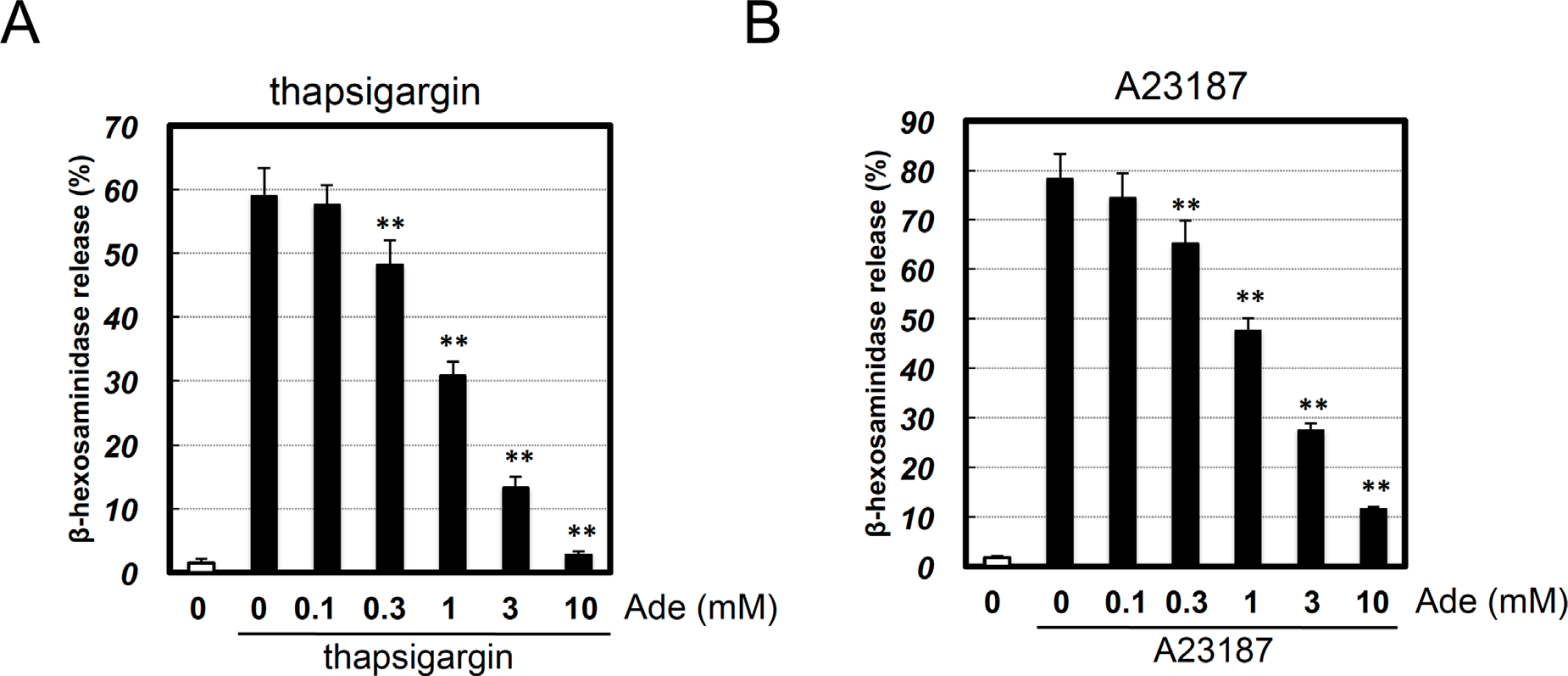
Adenine inhibited thapsigargin‐ or A23187‐induced degranulation. RBL‐2H3 cells were pre‐treated with adenine (Ade; 0.1–10 mM) for 30 min, and then stimulated with (A) thapsigargin (2 μM) or (B) A23187 (2.5 μM) for 15 min. Absorbance was measured with a microplate reader at 415 nm. *p < 0.05, **p < 0.01 versus the DNP‐BSA stimulus. *n* = 3.

An IgE/antigen stimulus increases the levels of IP_3_, which, in turn, acts on the endoplasmic reticulum and increases intracellular Ca^2+^ levels. This increase in Ca^2+^ levels is required for IgE/antigen‐induced degranulation. We confirmed the inhibitory effects of adenine on IgE/antigen‐induced degranulation (Fig. 1B), and subsequently investigated whether adenine inhibited degranulation evoked by Ca^2+^. Thapsigargin is an inhibitor of sarco‐endoplasmic reticulum Ca^2+^‐ATPases, which increase cytoplasmic Ca^2+^ concentrations. We treated RBL‐2H3 cells with thapsigargin and examined the effects of adenine on degranulation. We found that thapsigargin‐induced degranulation was dose‐dependently inhibited by adenine (Fig. 7A). A23187 is a Ca^2+^ ionophore that increases cytoplasmic Ca^2+^ concentrations by allowing ions to cross the cell membrane 20. We found that the treatment of cells with A23187 induced degranulation, and A23187‐induced degranulation was dose‐dependently inhibited by adenine (Fig. 7B). These results indicated that adenine inhibited degranulation induced by cytoplasmic Ca^2+^. On the other hand, the inhibitory effects of adenine on A23187‐ or thapsigargin‐induced degranulation were weaker than these of the IgE/antigen stimulus (compare Figs. 1B vs. 7AB). Thus, adenine may inhibit degranulation before and after increase in Ca^2+^ levels under the IgE/antigen stimulus.

### Adenine inhibited IKK α/β activation

**Figure 8:**
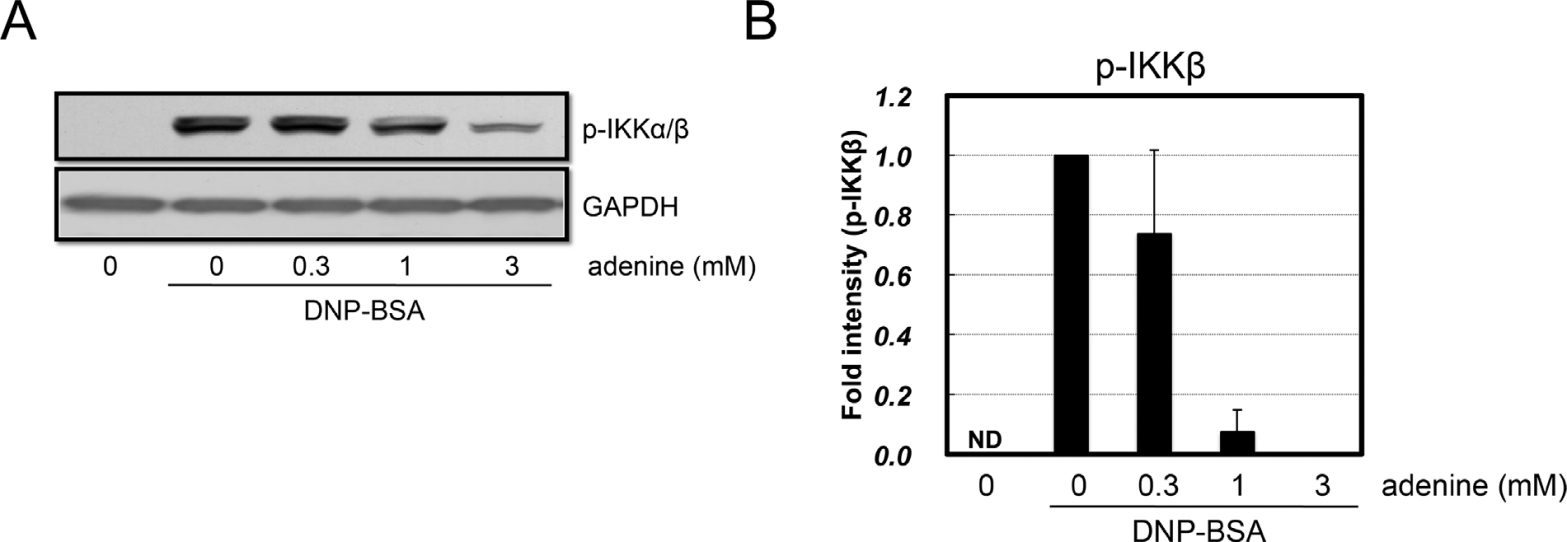
Adenine inhibited antigen‐induced IKKα/β phosphorylation. RBL‐2H3 cells were pre‐treated with adenine (0.3–3 mM) for 30 min, and then stimulated with DNP‐BSA (20 ng/mL) for 5 min. (A) A Western blotting analysis was performed using a specific antibody for phospho‐IKKα (Ser 180)/IKKβ(Ser 181). (B) A densitometric analysis of IKKα/β phosphorylation using image analyzing software. *n* = 3.

IKK β was previously shown to play a central role in degranulation by phosphorylating SNAP‐23 3. To elucidate the mechanisms responsible for the inhibitory effects of adenine on degranulation after increase in Ca^2+^ levels under the IgE/antigen stimulus, we measured the activation status of IKK. We found that the IgE/antigen stimulus enhanced IKK α/β phosphorylation, whereas adenine markedly attenuated it (Fig. 8). Therefore, the inhibitory effects of adenine on degranulation after increase in Ca^2+^ levels under the IgE/antigen stimulus may be mediated through IKK α/β.

## Discussion

Even though the adenine receptor has already been identified 9, the physiological role of adenine remains unclear. In the present study, we demonstrated that adenine negatively regulated IgE/antigen‐induced mast cell degranulation. In contrast with adenosine, which increases mast cell degranulation, adenine inhibited degranulation in the RBL‐2H3 mast cell line. Therefore, adenosine may enhance, whereas adenine may attenuate allergic reactions. These results provide an insight into the mechanisms underlying allergic responses. The structural difference between adenosine and adenine were shown (Fig. 1A). These differences may have influenced their effects on degranulation. On the other hand, purine, which does not have an amino group (Fig. 1A), did not inhibit degranulation. Therefore, the amino group of adenine may play an important role in inhibiting degranulation.

Syk plays an important role in mast cell degranulation, as Syk‐deficient mast cells fail to secrete leukotrienes and cytokines when stimulated by IgE/antigen 21, 22. In the present study, we demonstrated that adenine inhibited Syk as well as MAP kinase and PI3K/Akt signaling. Thus, the inhibitory effects of adenine on IgE/antigen‐induced degranulation and TNFα secretion may be mediated through Syk. These results are consistent with recently published report 23. On the other hand, these results contrast with the effects of adenosine, which has been shown to increase PI3K‐mediated signal transduction and degranulation 8. In preliminary experiments, the adenine receptor antagonist, PSB‐08162, was not able to reverse the inhibitory effect of adenine on the degranulation in RBL‐2H3 mast cells. These preliminary results suggest that another subtype of adenine receptors may be involved in the allergic response. Therefore, the mechanisms by which the adenine receptor inhibits degranulation, currently remain unknown and, thus, further studies are warranted. We herein demonstrated that adenine inhibited Ca^2+^ ionophore‐ or thapsigargin‐induced degranulation, suggesting that adenine also affected the degranulation process. SNAREs mediate the transport of secretory vesicles to the membranes of mast cells, which subsequently leads to exocytosis. A previous study reported that SNAP‐23, a t‐SNARE, was involved in exocytosis in mast cells 24. Furthermore, the IKK β‐mediated activation of SNAP‐23 was shown to be involved in degranulation 3. In the present study, we revealed that adenine inhibited IKK α/β activation under the IgE/antigen stimulus. Therefore, adenine may (1) inhibit FcϵRI‐mediated signal transduction by inhibiting Syk and (2) inhibit the degranulation process by inhibiting the activation of IKK α/β. These dual effects of adenine may have marked effects on anti‐allergic responses. We also observed the inhibitory effects of adenine in a mouse model of the PCA reaction. These results suggested that adenine played a physiologically important role by negatively regulating allergic responses in vivo. Adenine has been detected in healthy humans, and its levels were increased by chronic renal failure 25, 26. It is of interest to determine whether adenine or adenine derivatives are useful in ameliorating allergic responses in humans, which may shed light on the development of novel types of anti‐allergic drugs.

## Authors' Contributions

T.H., and K.O. designed research; T.H., S.I., F.O., K.T., M.Y., and M.K. performed research; T.H., S.I., F.O., K.T., M.Y., and M.K. analyzed data; C.M. provided reagent; T.H. wrote the paper.

## Acknowledgments

We thank Dr. Bellamkonda K. Kishore (University of Utah Health Sciences Center & Veterans Affairs Salt Lake City Health Care System) for the useful discussions. Animal experiments were supported by the Institute of Laboratory Animal Science (Hiroshima University).

## Conflicts of Interest

The authors declare no competing financial interests.

## Supporting information

Additional supporting information may be found in the online version of this article at the publisher's web‐site.

**Figure S1**. Adenine did not affect cell viability in RBL‐2H3 cells.
**Figure S2**. Effect of BSA on PCA reaction.
