# Supplementary material for: Mechanisms of the action of adenine on anti‐allergic effects in mast cells
Source: Immun Inflamm Dis. 2017 Nov 1;6(1):97–105. doi: 10.1002/iid3.200 (PMC5818451; doi:10.1002/iid3.200)
Supplement: Supplementary file 1 — Figure S1. Adenine did not affect cell viability in RBL‐2H3 cells. Figure S2. Effect of BSA on PCA reaction. [file IID3-6-97-s001.pdf]

Supplementary figures

Mechanisms of the action of adenine on anti-allergic effects in mast cells

Toru Hosoi[1], Shinsuke Ino[1], Fumie Ohnishi[1], Kenichi Todoroki[1], Michiko Yoshii[1], Mai Kakimoto[1], Christa E. Müller[2], and Koichiro Ozawa[1]

[1]Department of Pharmacotherapy, Graduate School of Biomedical and Health Sciences, Hiroshima University, 1-2-3 Kasumi, Minami-ku, Hiroshima 734-8551, Japan
Telephone/FAX: 81-82-257-5332
[2]PharmaCenter Bonn, Pharmaceutical Institute, Pharmaceutical Chemistry I, University of Bonn, An der Immenburg 4, D-53121 Bonn, Germany
Telephone: 49-228-73-2301 FAX: 49-228-73-2567

Supplementary methods

LDH leakage assay

Cell viability was analyzed using the Lactate Dehydrogenase Leakage Assay kit (Roche Molecular Biochemicals, Indianapolis, IN) according to the manufacturer's directions. LDH activity was measured at an optimal density of 492 nm.

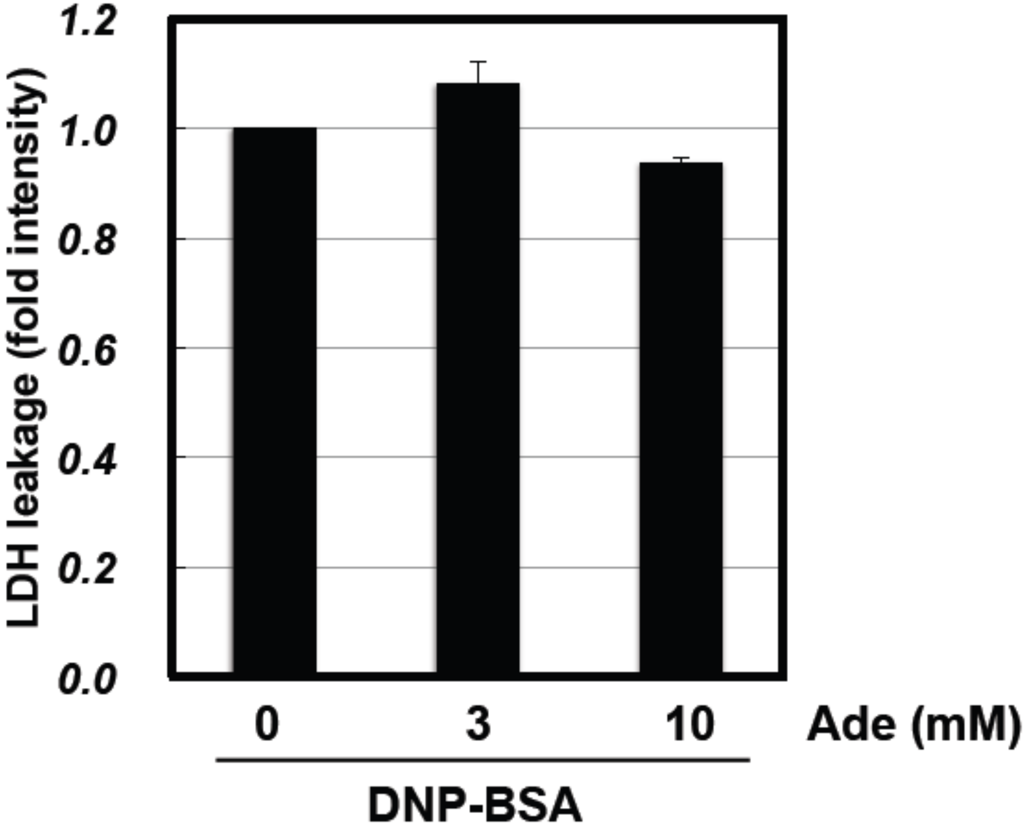

Sup Fig.1 Adenine did not affect cell viability in RBL-2H3 cells.
RBL-2H3 cells were pre-treated with adenine (Ade; 3, 10 mM) for 30 min, and then stimulated with DNP-BSA (20 ng/mL) for 15 min. The cytotoxicity of adenine on RBL-2H3 cells was determined by LDH assay.

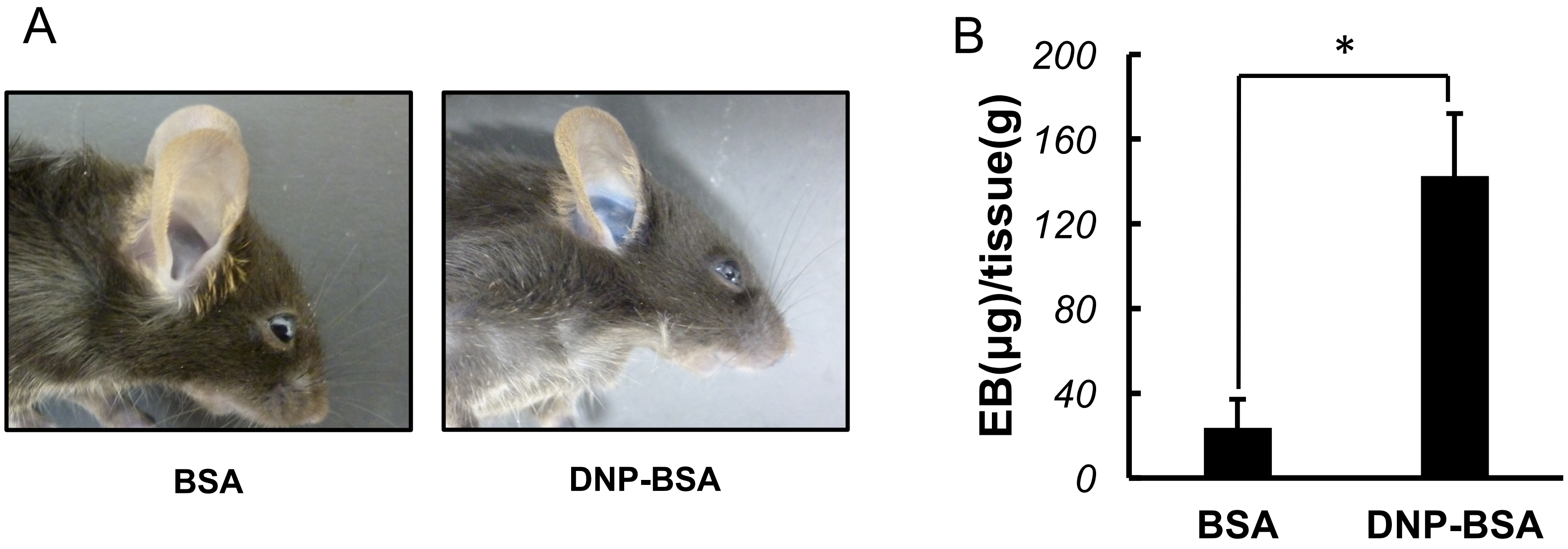

Sup Fig.2 Effect of BSA on PCA reaction.
(A) Compared to the effect of DNP-BSA plus Evans blue dye, BSA plus Evans blue dye injection did not cause the leakage of the dye. (B) The mass of Evans blue dye (μg) per g of ear tissue were calculated. *p<0.05, n=3-4
